# Multi-Dataset Comparison of Vision Transformers and Convolutional Neural Networks for Detecting Glaucomatous Optic Neuropathy from Fundus Photographs

**DOI:** 10.3390/bioengineering10111266

**Published:** 2023-10-30

**Authors:** Elizabeth E. Hwang, Dake Chen, Ying Han, Lin Jia, Jing Shan

**Affiliations:** 1 Department of Ophthalmology, University of California, San Francisco, San Francisco, CA 94143, USA; 2 Medical Scientist Training Program, University of California, San Francisco, San Francisco, CA 94143, USA; 3 Digillect LLC, San Francisco, CA 94158, USA

**Keywords:** glaucoma, deep learning, vision transformer, fundus photography

## Abstract

Glaucomatous optic neuropathy (GON) can be diagnosed and monitored using fundus photography, a widely available and low-cost approach already adopted for automated screening of ophthalmic diseases such as diabetic retinopathy. Despite this, the lack of validated early screening approaches remains a major obstacle in the prevention of glaucoma-related blindness. Deep learning models have gained significant interest as potential solutions, as these models offer objective and high-throughput methods for processing image-based medical data. While convolutional neural networks (CNN) have been widely utilized for these purposes, more recent advances in the application of Transformer architectures have led to new models, including Vision Transformer (ViT,) that have shown promise in many domains of image analysis. However, previous comparisons of these two architectures have not sufficiently compared models side-by-side with more than a single dataset, making it unclear which model is more generalizable or performs better in different clinical contexts. Our purpose is to investigate comparable ViT and CNN models tasked with GON detection from fundus photos and highlight their respective strengths and weaknesses. We train CNN and ViT models on six unrelated, publicly available databases and compare their performance using well-established statistics including AUC, sensitivity, and specificity. Our results indicate that ViT models often show superior performance when compared with a similarly trained CNN model, particularly when non-glaucomatous images are over-represented in a given dataset. We discuss the clinical implications of these findings and suggest that ViT can further the development of accurate and scalable GON detection for this leading cause of irreversible blindness worldwide.

## 1. Introduction

Glaucoma is a group of chronic, progressive optic neuropathies are a leading cause of vision loss worldwide [1]. Primary open-angle glaucoma (POAG) is the most common type of glaucoma, with cases estimated to rise from 2.7 million in 2011 to 7.3 million by 2050 in the United States alone [2]. While most often associated with increased intraocular pressure (IOP), the disease process can also occur with normal or low IOP and is often referred to as the “silent thief of sight” because it typically progresses slowly and without noticeable symptoms in its early stages. Thus, early detection, close monitoring, and timely interventions are key to preserving vision in glaucoma patients, especially among minority populations such as Hispanics/Latinos and African Americans, who are disproportionately affected relative to non-Hispanic Whites [3]. However, currently the United States Preventive Services Task Force (USPSTF) does not recommend screening for primary open-angle glaucoma in asymptomatic adults 40 years or older. In their updated 2022 review, the USPSTF cited the need for targeted screening among high-risk populations (such as individuals with a family history of glaucoma or from disproportionately affected minority groups), optimizing contemporary screening approaches and modalities to improve both efficiency and cost-effectiveness, and clinical trials demonstrating the utility of such screening approaches in vision-related patient outcomes [4].

Deep learning-aided diagnostic interpretation has received significant interest for its potential to improve the accuracy of diagnosing glaucoma and deliver high-throughput screening tools optimized for early diagnosis in at-risk patients [5]. Glaucoma diagnosis often requires complex medical imaging of the optic nerve and retina in a specialist setting, and even then, is subject to inter-observer variability. Deep learning models have the potential to detect subtle structural changes missed by the eye, provide consistent results, and improve efficiency by reducing the burden on glaucoma specialists. Application of deep learning models to glaucoma diagnosis would also allow for high-throughput screening to identify asymptomatic disease and improve patient outreach, particularly in resource-limited settings. 

Among the imaging modalities, fundus photography is a widely available, relatively low-cost approach already employed for clinical use in diabetic retinopathy tele-screening. In glaucoma, fundus photos provide the vertical optic nerve cup-to-disc ratio (vCDR), which quantifies the relationship between the cup (the central depression on the optic nerve head) and the disc (the entire optic nerve head) which enlarges as the disease progresses. Interpretation of these photos, however, can be difficult to reproduce among even expert specialists, and exhibit high rates of inter-observer variability [6,7,8], as well as being subject to observer bias (e.g., the tendency to under-call optic neuropathy in small optic discs while overcalling disease in physiologically large discs [9]). Therefore, the development and application of an AI tool to classify GON could greatly enhance fundus photography’s utility as a population-based screening tool. 

Previous studies have shown that deep learning models individually trained on color fundus photos [10], visual field analysis [11,12,13,14], and optical coherence tomography (OCT) [15,16,17,18,19] are able to identify glaucomatous optic neuropathy (GON) with robust performance (comparisons of specific deep learning models developed for glaucoma diagnosis and discussions of the different approaches are thoroughly covered in excellent reviews from Thompson et al. [5] and Yousefi [20]). Indeed, a recent meta-analysis of 17 deep-learning models trained on diagnosing GON from fundus photographs reported an overall AUC of 0.93 (95% CI 0.92–0.94), slightly lower than the AUC reported for studies using OCT (overall AUC 0.96, 95% CI 0.94–0.98) [21]. Several of these studies included external validation sets of up to six cohorts, suggesting that their models may generalize to unseen outside data. However, because these models are large and require intensive computational resources to train, they have been trained on datasets that are most often inaccessible to the public, thus making it difficult to compare whether the models themselves show differences during the training process.

To date, many AI models for glaucoma classification have utilized convolutional neural networks (CNNs). CNNs provide a scalable approach to object recognition within images by processing spatial patterns and extracting relevant features [22]. This architecture enables CNNs to automatically learn hierarchical representations of the features in an image. In supervised learning, CNNs are trained on labeled datasets, while in unsupervised learning, unsupervised methods like autoencoders are utilized for feature extraction. Semi-supervised learning, such as transfer learning, are also commonly described, as pre-trained models can be fine-tuned with smaller labeled datasets to improve performance [5,23]. However, a well-known attribute of CNNs is their inherent bias towards translation-invariant object recognition [24] which permits the interpretation of features outside of their spatial context [25], leaving models vulnerable to artifactual errors. Current models attempt to alleviate this by strict standardization of inputs, which, unfortunately, further restricts the ability of CNN algorithms to generalize to new, and even related, tasks without labor-intensive preprocessing.

In the last decade, Vision Transformer (ViT) [26], among other transformer architectures [27], has taken advantage of the self-attention mechanisms used in natural language processing to improve upon these limitations of CNNs. In contrast to CNNs, ViT models process the entire image as a sequence of patches, thus allowing for the capture of global relationships. ViTs have also been shown to generalize from smaller datasets than CNNs, which are heavily reliant upon pre-training and fine-tuning for optimal performance [26]. ViT models have now been applied to the analysis and interpretation of a wide range of clinical data ranging from electrocardiograms [28] to intraoperative surgical techniques [29]. In ophthalmology, there are increasing reports of ViT models trained to classify retinal pathologies from fundus photography [30,31,32] and OCT imaging [33,34,35,36], including several assessing their performance relative to CNNs [31,34,35]. Given that glaucoma diagnosis often requires multimodal imaging that correlates structural and functional data, it has been theorized that the global attention mechanisms utilized by ViTs offer an advantage over CNNs’ dependency upon local features. However, few such reports in the ophthalmic literature benchmark one model against the other, and even fewer compare the outcomes from more than one training dataset. This represents a knowledge gap for AI-guided GON detection, since an optimal architecture should be able to generalize across variables that vary by clinical setting, such as patient population, image format, and disease prevalence.

In this report, we describe the training of ViT and CNN models on six publicly available, independent datasets, compare the two models’ accuracies, and discuss the potential clinical applications for each type of model. We propose that the choice between these two model architectures may depend upon the specific clinical setting, labeled data availability, and computational resources. Ultimately, we hope that our results provide insight into model selection for specific clinical tasks as well as effective database construction.

## 2. Materials and Methods

### 2.1. Datasets

**Figure 1:**
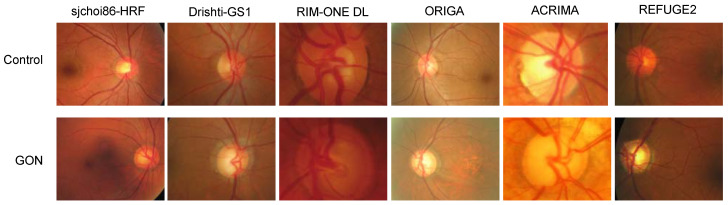
Representative fundus photographs from the datasets used in this study. GON: glaucomatous optic neuropathy.

A total of six public datasets were included for analysis in this paper (Figure 1, Table 1) [37,38,39,40,41,42]. Complete dataset sizes varied between 101 images (Drishti-GS1) to 720 images (REFUGE2). Though representation of non-glaucomatous (control) and glaucomatous classes varied between the datasets, no obvious correlation existed between total dataset size and class ratios (Table 1). When provided by the original authors, the patient selection criteria and instrument cameras are also noted in Table 1.

All datasets included ground truth labels indicating GON or control. Most datasets derived ground truth from expert labeling and clinical annotations with the exceptions of ORIGA (algorithm-based) and sjchoi86-HRF (unknown). Three datasets (sjchoi86-HRF, ORIGA, and REFUGE2) provided whole fundus images, one provided OD-centered images (Drishti-GS1), and two provided OD-cropped images (RIM-ONE DL and ACRIMA) (Figure 1). Sources accessed for each of the datasets are provided in the references. No photographs were excluded from our analysis.

### 2.2. Image Preprocessing

**Figure 2:**
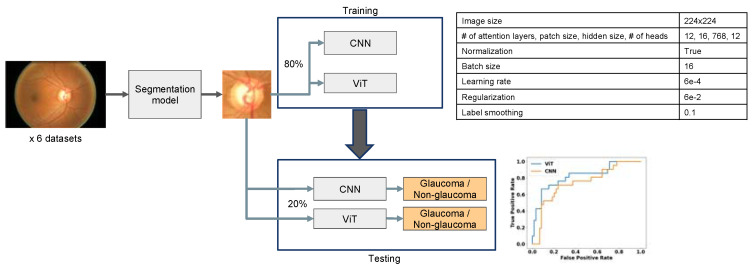
Workflow of ViT vs. CNN model training (with hyperparameters) and validation.

To minimize the presence of redundant information which could potentially impact deep learning model performance, we conducted pre-processing of all images to extract the region around the optic nerve head from each fundus image as shown in the depicted model (Figure 2). This was achieved using deeplabv3plus [43], a semantic segmentation model. Once the region of interest was extracted, we automatically cropped a square area centered around the disc. These extracted images were then utilized to train the CNN and ViT models described below. By focusing on specific areas, we aimed to improve the model’s ability to identify glaucoma-related features and enhance the accuracy of the automated detection system.

### 2.3. Vision Transformer (ViT) and ResNet Training and Evaluation

Each one of the public databases was split into a training set (80%) and a testing set (20%) (Figure 2). This ensured a consistent and fair evaluation of both models using identical testing datasets. An overview of our method is shown in Figure 2. For the CNN model, we leveraged the standard ResNet-50 which has 50 layers with incorporated residual connections with no further tuning [43]. For the ViT architecture [25], we used 12 attention layers and a patch size of 16, hidden size of 768, and 12 heads. Following the practices established by [44] and [25], we pretrained the ViT on the ImageNet dataset [45]. The images were resized to a uniform size of 224 × 224 pixels. Additionally, we normalized the pixel values to a range between 0 and 1. During training, we used a batch size of 16 and employed the AdamW optimizer with a learning rate of 6e^-4^ and regularization of 6e^-2^. These hyperparameters (included in Figure 2) were chosen to optimize the model’s convergence and performance. To compute the loss during training, we employed cross-entropy loss with 0.1 label smoothing.

Performance metrics, including area under the receiver operating characteristic curve (AUC), sensitivity, specificity, accuracy, F1 score, and mAP (mean Average Precision), were calculated from models evaluated on the held-out test sets (Table 2). Specificities were calculated at a fixed sensitivity threshold of 50%. Confidence intervals (CI) were determined by bootstrap resampling of the test sets with replacement (*n* = 10 times) while the training sets and models remained fixed.

## 3. Results

**Figure 3:**
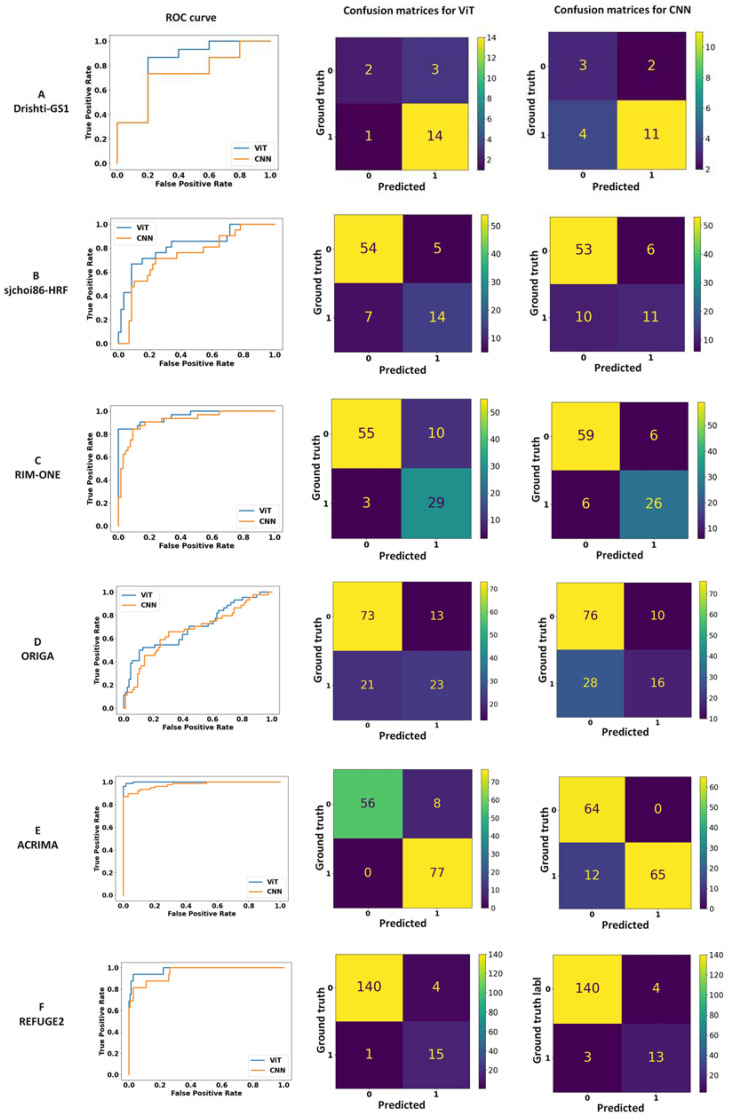
ROC curves and confusion matrices for ViT and CNN models trained on individual datasets (**A**–**F**). For the confusion matrices, a classification of 0 refers to control/non-glaucomatous, whereas a classification of 1 refers to glaucomatous. Ground truth labels were used as provided by the original datasets (ref. Table 1).

In Table 2 and Figure 3, we present the performance statistics and contingency tables of the CNN and ViT models trained to classify non-glaucomatous (non-GC) from glaucomatous (GC) eyes on each of the six public datasets. When compared using relative AUC, the ViT models were non-inferior to the CNN models and appeared to outperform the CNN models on five of the six datasets, though this was not statistically significant given the overlapping confidence intervals. The greatest differences were observed among the sjchoi86-HRF (0.79 ViT vs. 0.71 CNN), ORIGA (0.69 ViT vs. 0.62 CNN) and REFUGE2 (0.95 ViT vs. 0.89 CNN) datasets. No difference in mean AUC was observed for only one dataset, Drishti-GS1 (both 0.67). The performance on the remaining two datasets were also consistently, if marginally, higher for the ViT models (RIM-ONE: 0.88 ViT vs. 0.86 CNN; ACRIMA 0.94 ViT vs. 0.92 CNN). Similar observations were made for the accuracies, F1 scores, mAP, as reflected in the average statistic and the 95% confidence intervals.

The recall or sensitivity of the ViT models surpassed those of the CNN models among the six datasets by an average of 0.14, with the largest difference observed in the Dristhi-GS1 (0.93 ViT vs. 0.73 CNN) and the smallest in REFUGE2 (0.94 ViT vs. 0.81 CNN). By contrast, the specificities were more varied between the two methods, ranging from comparable (sjchoi86-HRF: ViT 0.92 vs. CNN 0.90; ORIGA: 0.85 ViT vs. 0.88 CNN; REFUGE2: ViT 0.97 vs. CNN 0.97) to favoring the CNN model (RIM-ONE: 0.85 ViT vs. 0.91 CNN; ACRIMA: 0.88 ViT vs. 1.00 CNN). For Drishti-GS1, the small sample size of non-GC images in the held-out test set (*n* = 5 images) resulted in inconclusive specificity statistics as reflected by the 95% confidence intervals of (0,1) for both models.

We noted that ViT tended call more false positives (i.e., label control images as GON) than CNN models in several datasets, including RIM-ONE (10 ViT false positives (FP) vs. 6 CNN FP), ORIGA (13 ViT FP vs. 10 CNN FP), and ACRIMA (8 ViT FP vs. 0 CNN FP). Accordingly, two of these ViT models demonstrated lower specificities than their CNN equivalents (i.e., RIM-ONE: 0.74 ViT vs. 0.81 CNN; ACRIMA: 0.91 ViT vs. 1.00 CNN).

**Figure 4:**
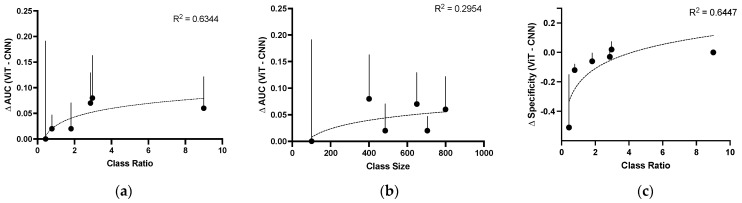
ViT outperforms CNN models in datasets with greater class imbalance but not class size. (∆ = ViT − CNN, where ViT outperforms CNN when ∆ > 0, and CNN outperforms ViT when ∆ < 0) Log-linear regression models (dotted lines) are included with coefficients of determination as indicated. (**a**) ∆AUC as a function of class ratio. (**b**) ∆AUC as a function of class size. (**c**) ∆Specificity as a function of class ratio. See Table 1 for class sizes and ratios.

Interestingly, ViT outperformed CNN on datasets with higher ratios of non-GC to GC photos (Figure 4a), though not with total dataset size (Figure 4b). This was most clearly evidenced by the delta AUC of the Drishti-GS1 and REFUGE2 models, whose datasets harbored the lowest (0.44) and highest (9.0) ratios of non-GC to GC images, respectively. Furthermore, the differences in specificity between the ViT and CNN models diminished as the ratio of non-GC to GC images increased (Figure 4c): when trained on the REFUGE2 dataset, the specificity of the ViT model overlapped with that of the CNN model (0.97, CI 0.94–0.99).

## 4. Discussion

Here we focus the performance of ViT and CNN models trained on glaucoma detection from a single imaging modality, fundus photography. We take advantage of ImageNet pre-trained models to test and train each architecture on six publicly available annotated datasets, which were collected from at least four countries (India, Spain, Singapore, and China) and varied in size from 101 to 800 total images. Class imbalances between control and glaucomatous labeled images were present to varying degrees among the datasets, from the most evenly matched (Drishti-GS1, class ratio of 0.44 or 69% glaucoma prevalence) to the least (REFUGE2, class ratio of 9.0 or 10% glaucoma prevalence). A survey of 14 US-based studies found all glaucoma prevalence rates ranging from 2.1% to 25.5%, and POAG prevalence rates between 1.86% and 13.8% [44]. Thus, though these datasets represent selected cohorts rather than a population survey, it is likely that the “lower” prevalence cohorts more accurately reflect the dataset composition that would be expected from a moderate to high-risk screening population. We had two objectives from comparing the models trained upon multiple, rather than pooled, datasets: first, to ask whether the ViT model could perform equal to, or better than, a widely accepted CNN model, ResNet-50, and second, to determine whether ViT or CNN models, when trained on datasets of different sizes and class representations, demonstrated any trends in performance metrics including AUC, sensitivity, and specificity, that might inform future clinical application of the two architectures.

As predicted, we found that the pre-trained ViT model matched or outperformed the equivalent CNN model on all six datasets by AUC and accuracy measures. We also observed that the ViT models increasingly outperformed CNN models, as measured by AUC and specificity, on datasets with greater representations of controls (i.e., higher class ratio). We suggest that this difference may reflect that, when presented with insufficient control representation, ViT struggles with the greater variability present among non-glaucomatous optic nerve discs due to the wider array of potential relationships when using a global attention mechanism. However, as the ViT algorithm is presented with an increasing number of control examples, it can better assign global relationships to a given class, even when it is as varied as “non-glaucoma”.

Given our observations, we would recommend CNN models for GON detection in tasks with uniform data collection where high test specificity outweighs other considerations. In contrast, we would nominate ViT models for tasks requiring collaborative data collections (e.g., clinical trials, multi-site tele-screening), whereby different operators, patient demographics, camera models, and data processing standards are likely to result in datasets with levels of heterogeneity beyond that which CNN models can accommodate. ViT performance could be enhanced further by targeted deployment to patients with identifiable risk factors, such as a family history of glaucoma, advanced age, or predisposing conditions such as steroid use. Such at-risk populations exhibit higher pre-test probability and would thus benefit from ViT’s greater sensitivity.

While CNN and ViT models are widely utilized for high-throughput image analysis and classification, their differences in feature detection and training requirements have led to the suggestion that ViT models may improve upon CNN performance. CNN architecture utilizes a sliding window method to extract features in a local fashion and thus has strict input requirements [45]. Previous strategies for improving CNN performance of photographic GON detection have focused on the optimization of pre-processing techniques like data augmentation [46] and feature extraction [47], as well as more clinically motivated strategies such as structure-function correlation between multiple testing modalities [48,49]. More recently, transformer architectures have gained interest for their ability to use global attention mechanisms to identify long-range interactions [50] and their flexibility in allowing for non-uniform inputs [26]. Therefore, while the prevalence of inductive biases in CNNs relative to transformers may enable ResNet models to outperform ViT models when classification relies upon the presence or absence of locally identifiable features (e.g., optic nerve thinning in defined superior-temporal or inferior-nasal patterns) [26,51]. ViT may ultimately offer superior performance when diagnostic features are distributed in a disconnected manner (e.g., identifying glaucomatous features such as bayoneting). This would be particularly applicable in the setting of multimodal imaging datasets that could potentially rely upon global features, such as the correlation of functional visual field testing with structural changes in the OCT, which so far have required a multi-algorithmic approach [52].

Yet, despite the potential for transformers to incorporate long-range feature detection from multimodal datasets, the literature comparing ViT models to CNN models have generally focused on single modalities due to the challenges of multimodal data integration as an input into a single algorithm [31,32,35,51,52,53,54,55]. One outstanding report compares ViT to CNN models trained on Diabetic Retinopathy (DR) detection from multiple independent datasets consisting of either fundus photos or OCT imaging, and finds that ViT models are superior in both cases; however, no multimodal datasets are used [31]. To the best of our knowledge, only two publications so far have compared the performance of published ViT and CNN models on glaucoma detection from fundus photos [51,56]. In one report, the authors found that Data-efficient Image Transformer (DeIT) models outperformed similarly trained ResNet-50 models [51]. They further compared the DeIT attention maps with ResNet-50 average saliency maps to demonstrate that the transformer model more precisely focused upon the borders of the optic disc where glaucomatous features are most often identified, whereas the CNN saliency maps highlighted the entire optic nerve. Intriguingly, the more recent report found that the ViT model underperformed the CNN models (VGG, ResNet, Inception, MobileNet, DenseNet) on an external validation set [56]. While not directly comparable to our results, we note that their training set was also comprised of three nearly equally represented classes (GON, non-GON, and normal optic discs), perhaps resembling our “lower” class ratio datasets, such as ACRIMA.

Our work builds upon these studies by incorporating the use of independent training sets similar to [31] as well as avoiding the use of fine-tuning between datasets, thus allowing for observations on the baseline performance of the two architectures in multiple settings. Within the constraints of the public datasets utilized by our models, our results suggest that simply switching to ViT-based architecture alone will not significantly improve model performance. This is for two reasons: First, though there is an appreciable trend of higher mean AUCs across the ViT models, the differences between the individual ViT and CNN models were not statistically significant. Second, while ViT models uniformly demonstrated greater sensitivities than the CNN models, we observed that the under-representation of non-GC images during training may have led to lower model specificities. This implies that one trade-off of ViT’s global attention mechanism may result in increased dependence upon sufficient class representation during training, which aligns with previous observations that, for smaller datasets, ViT-based architectures are more dependent upon training set representation than CNN-based architectures [26]. Thus, improving model performance may not rely only upon optimizing the model itself, but also the training data and processes involved. Here we utilized pre-trained models, but other techniques to improve model performance have included transfer learning [57], artifact-tolerant feature representation [10], cross-teaching between CNN and transformer models [58], and hybrid CNN-ViT architectures which extract local features in a patch-based manner [55]. While not addressed here, many of these strategies appear promising and merit further investigation.

We acknowledge a couple of limitations in our study. First, our comparisons of the two models were limited to datasets containing only fundus photography, while in practice, the gold standard diagnosis of glaucomatous optic neuropathy requires the correlation of structural findings (optic nerve thinning) with functional ones (visual field defects) [45]. Secondly, we pre-processed the fundus photos with optic nerve head segmentation to avoid biasing the models with non-disc-related information. Given that ViT uses a global mechanism, we anticipate that the performance of the ViT models may have been disproportionately affected relative to the CNN models. However, given that real-world application of these models often incorporates similar pre-processing for a variety of reasons [59,60], we suggest that this approach remains relevant to clinical practice.

Future works based on these findings may benefit from comparisons of CNN-based vs. ViT-based models on larger cohorts, more rigorous investigations of whether non-glaucomatous representation impacts model performance, and ideally, side-by-side comparisons of both models in various clinical contexts (i.e., screening vs. specialty visits) to determine their efficacies and practicalities in different settings.

## 5. Conclusions

Overall, our findings suggest that ViT-based algorithms show excellent results regarding glaucoma detection in line with previous studies. However, our results indicate that, despite recent publication trends, CNN models may offer advantages over Transformer models for training datasets with more equal representation of both non-glaucomatous and glaucomatous images. For high-risk populations or other situations where the importance of detecting any disease outweighs the risk of false positives, we propose that ViT models should be considered superior to the more widely utilized CNN-based architectures established within the field.

Automated image processing algorithms for the detection of glaucomatous optic neuropathy can empower population-based screening towards preventing irreversible vision loss. We hope our findings here can further the development of accurate and scalable high-throughput methods for this leading cause of blindness worldwide.

## Figures and Tables

**Table 1:** Characteristics of publicly available datasets used in this study, as ordered by total set size.

Study	Patient Selection	Instrument	Ground Truth	Images			
				Non-GC (Control)	GC	Total Size	Class Ratio *
Drishti-GS1 [37]	Glaucomatous and routine refraction images selected by experts from patients between ages 40 and 80 at Aravind Eye Hospital in India	Not noted	4 experts	31	70	101	0.44
sfchoi86-HRF [38]	Unknown	Unknown	Unknown	300	101	401	2.97
RIM-ONE DL [39]	Curated extraction from RIM-ONE V1, V2, and V3 of glaucomatous and healthy patients from 3 hospitals in Spain	V1/V2: Nidek AFC-210 camera V3: Kowa WX 3D non-stereo camera	2 experts with tiebreaker	312	173	485	1.80
ORIGA [40]	Glaucomatous and randomly selected non-GC images from cross-sectional population Singaporean study (SiMES) of Malay adults between ages 40 and 80	Canon CR-DGi	ORIGA-GT	482	168	650	2.87
ACRIMA [41]	Glaucomatous and normal images selected by experts in Spain based on clinical findings	Topcon TRC retinal camera	2 experts	309	396	705	0.78
REFUGE2 [42]	Random selection from glaucoma and myopia study cohorts in China (Zongshan Ophthalmic Center)	KOWA, TOPCON	7 experts	720	80	800	9.00

* Calculated as a ratio of non-GC: GC images. GC = glaucomatous.

**Table 2:** Performance statistics for ViT and CNN models evaluated on held-out test sets. Confidence intervals of 95% are reported in parentheses.

	ViT			CNN		
	*AUC*	*Sensitivity*	*Specificity*	*AUC*	*Sensitivity*	*Specificity*
**Drishti-GS1**	0.67	0.93	0.40	0.67	0.73	0.91
	(0.44, 0.97)	(0.79, 1.00)	(0.00, 1.00)	(0.38, 0.91)	(0.50, 0.93)	(0.00, 1.00)
**sjchoi86-HRF**	0.79	0.67	0.92	0.71	0.52	0.90
	(0.67, 90)	(0.46, 0.82)	(0.84, 0.98)	(0.59, 82)	(0.31, 0.75)	(0.81, 0.97)
**RIM-ONE DL**	0.88	0.91	0.85	0.86	0.81	0.91
	(0.81, 0.94)	(0.79, 1.00)	(0.76, 0.93)	(0.78, 0.93)	(0.67, 0.94)	(0.83, 0.97)
**ORIGA**	0.69	0.52	0.85	0.62	0.36	0.88
	(0.60, 0.77)	(0.37, 0.67)	(0.77, 0.92)	(0.54, 0.70)	(0.21, 0.52)	(0.81, 0.95)
**ACRIMA**	0.94	1.00	0.88	0.92	0.84	1.00
	(0.90, 0.97)	(1.00, 1.00)	(0.79, 0.95)	(0.88, 0.96)	(0.76, 0.92)	(1.00, 1.00)
**REFUGE2**	0.95	0.94	0.97	0.89	0.81	0.97
	(0.88, 1.00)	(0.80, 1.00)	(0.94, 0.99)	(0.78, 0.99)	(0.60, 1.00)	(0.94, 0.99)
	**ViT**			**CNN**		
	***Accuracy***	***F1 Score***	***mAP***	***Accuracy***	***F1 Score***	***mAP***
**Drishti-GS1**	0.80	0.87	0.82	0.70	0.79	0.82
	(0.60, 0.95)	(0.73, 0.97)	(0.63, 0.99)	(0.50, 0.90)	(0.58, 0.93)	(0.61, 0.98)
**sjchoi86-HRF**	0.81	0.68	0.53	0.80	0.58	0.46
	(0.72, 0.89)	(0.51, 0.82)	(0.35, 0.72)	(0.71, 0.89)	(0.37, 0.76)	(0.28, 0.65)
**RIM-ONE DL**	0.87	0.82	0.70	0.88	0.81	0.72
	(0.79, 0.93)	(0.71, 0.90)	(0.56, 0.85)	(0.80, 0.94)	(0.69, 0.91)	(0.56, 0.86)
**ORIGA**	0.74	0.57	0.50	0.71	0.46	0.44
	(0.66, 0.82)	(0.44, 0.69)	(0.37, 0.63)	(0.63, 0.78)	(0.31, 0.60)	(0.32, 0.56)
**ACRIMA**	0.94	0.95	0.91	0.91	0.92	0.93
	(0.91, 0.98)	(0.91, 0.98)	(0.84, 0.96)	(0.87, 0.96)	(0.86, 0.96)	(0.89, 0.97)
**REFUGE2**	0.97	0.83	0.72	0.96	0.79	0.64
	(0.94, 0.99)	(0.64, 0.95)	(0.47, 0.92)	(0.93, 0.99)	(0.60, 0.93)	(0.39, 0.86)

## Data Availability

Publicly available datasets were analyzed in this study.
